# The impact of smoking on periodontitis patients’ GCF/serum cytokine profile both before and after periodontal therapy: a meta-analysis

**DOI:** 10.1186/s12903-023-02768-8

**Published:** 2023-02-01

**Authors:** Chun-Ping Hao, Nan-Jue Cao, Yu-He Zhu, Wei Wang

**Affiliations:** School and Hospital of Stomatology, China Medical University, Liaoning Provincial Key Laboratory of Oral Diseases, Shenyang, Liaoning People’s Republic of China

**Keywords:** Smoking, Periodontitis, Periodontal therapy, Cytokines, Immune response

## Abstract

**Background:**

Smoking is an established modifying factor for the host immune response of periodontitis patients. However, its exact influence remains unclear. We aimed to compare the cytokine profile of periodontitis patients with and without smoking habits both before and after periodontal therapy to preliminarily explore its influence on the host immune response to periodontitis.

**Methods:**

The protocol of the present meta-analysis was registered in the International Prospective Register of Systematic Reviews (PROSPERO) under the code CRD42021255656. Meta-analysis was performed for each cytokine if at least three studies were included. We synthesized the evidence to compare the cytokine profile of periodontitis with and without smoking both in gingival cervical fluid (GCF) and serum to explore the impact of smoking on periodontitis both locally and systemically. Moreover, we also compared the cytokine profile of the two groups of patients after periodontal therapy to explore the effect of smoking on the outcome of periodontal therapy.

**Results:**

Fifteen studies were included in this meta-analysis. We found that there was no significant difference between the two groups of patients in the baseline cytokine profile. However, after periodontal therapy, smoking periodontitis patients showed significantly higher IL-1β levels in their GCF than nonsmoking patients.

**Discussion:**

There was no significant difference between smoking and nonsmoking periodontitis patients in the baseline cytokine profile. However, after periodontal therapy, smoking periodontitis patients showed significantly higher IL-1β levels in their GCF than nonsmoking patients, which indicates that smoking may impair the response of periodontitis to periodontal treatment.

**Supplementary Information:**

The online version contains supplementary material available at 10.1186/s12903-023-02768-8.

## Introduction

Periodontitis is a public health concern worldwide [1]. Severe periodontitis can induce alveolar bone absorption and tooth loss, causing adverse impacts on both physical and mental health [2, 3]. Its mechanism has been widely researched with the goal of finding a valid therapy method [4]. Although the consensus is that microbial infection is the initiator of periodontitis, the role of the host immune response to pathogenic bacteria has gained attention [5, 6]. Host immunologic responses can protect host tissue against bacterial attack, while in sensitive hosts, the immune response may be overreactive, causing tissue damage [7]. Studies have found that host susceptibility and the immune response are decisive factors in the progression of periodontitis [8].

Smoking has been recognized as a factor promoting the progression of periodontitis in the new periodontitis classification proposed by the 2018 world workshop [9]. Periodontitis patients with smoking habits often show worse symptoms and a higher risk of progression than nonsmoking patients [10, 11]. In addition, significantly less bleeding on probing (BOP) was observed in smoking periodontitis patients than in nonsmoking patients, which may mask gingival inflammation and delay therapy [12, 13]. Moreover, smoking may have adverse effects on the success of periodontal treatment [14, 15].

Due to the strong modifying effect of smoking on periodontitis, its mechanism has attracted a lot of interests from researchers [16]. One widely recognized mechanism is that smoking could damage the host immune defence. Smoking modifies both innate and adaptive immunity. For innate immunity, smoking reduces the chemotaxis and phagocytosis function of neutrophils [17, 18]. For adaptive immunity, smoking disturbs Th1/Th2/Th17 immune homeostasis [19, 20]. Moreover, smoking could activate important inflammatory-related molecular mechanisms, such as nuclear factor kappa B (NFκB) and mitogen activated protein kinase (MAPK), to regulate the immune response [21, 22].

Cytokines are mediators of the inflammatory/immunologic process [23–25]. The level of cytokines could reflect the state of the host immune response [26]. Many studies have been devoted to determining the modifying effect of smoking on the level of cytokines in periodontitis [27]. Some studies have found that smoking periodontitis patients showed elevated levels of proinflammatory cytokines, such as IL-1β and TNF-α, compared with nonsmoking periodontitis patients [28, 29]. Moreover, smoking could shift the ratios of proinflammatory/anti-inflammatory cytokines of periodontitis patients to a more proinflammatory state [20]. However, other studies have found that there is no significant difference in the level of cytokines between periodontitis patients with and without smoking [30]. The exact influence of smoking on the cytokine profile of periodontitis remains unclear.

In this meta-analysis, we aimed to determine whether smoking could modify the cytokine profile of periodontitis patients. Considering that smoking is reported to compromise the effect of periodontal therapy, we also synthesized evidence to explore the relationship between smoking and cytokine profiles in periodontitis patients after periodontal therapy [31]. To explore the effect of smoking on periodontitis patients locally and systematically, we explored the cytokine profile both in gingival crevicular fluid (GCF) and serum. This meta-analysis preliminarily explored the effect of smoking on periodontitis patients’ immune response in terms of cytokine profiles. This study could provide evidence to understand better the mechanism underlying the effect of smoking on the immune response to periodontitis.

## Methods

This meta-analysis was conducted according to the Preferred Reporting Items for Systematic Review and Meta-Analysis (Additional file 1: PRISMA) guidelines [32]. The protocol of the current meta-analysis was registered in the International Prospective Register of Systematic Reviews (PROSPERO) under the code CRD42021255656 (https://www.crd.york.ac.uk).

### Focused questions

P (population): Patients with periodontitis;

I (intervention): Cigarette smoking;

C (comparison): Nonsmoking;

O (outcome): GCF/serum cytokine profile both before and after periodontal therapy;

Research question: The effect of smoking on the GCF/serum cytokine profile of periodontitis patients both before and after periodontal therapy.

### Search strategy

We searched Medline (PubMed), Cochrane, EMBASE, and Web of Science to identify studies published through April 2021. Moreover, we updated the search results in April 2022. There was no restriction on article language. The search strategy was designed based on the combination of the following key words: “smoke”, “smoking”, “smoking habit”, “periodontitis”, “chronic periodontitis”, “periodontal diseases”, “cytokines”, “biomarkers”, “immune markers”, “chemokines”, and so on. The detailed search strategies used for all databases are presented in the Additional file 2.

### Selection criteria

The eligibility criteria were as follows: (1) chronic periodontitis patients without periodontal therapy history and systemic diseases/conditions; (2) studies reporting the cytokine profile in GCF and (or) serum; (3) studies reporting the concentration of cytokines and (or) total amounts were included; (4) studies reporting results with proper statistical methods, such as the mean ± standard deviation (SD), were included; (5) cross-sectional studies, case–control studies, cohort design studies, and randomized controlled trials (RCTs) were included; and (6) since studies focusing on patients with systemic diseases/conditions may also have healthy patients as control groups, these studies were also screened to find potential eligible healthy controls.

The exclusion criteria were as follows: (1) reviews; (2) patients with chronic periodontitis who had already been treated, such as scaling and root planing (SRP) and local/systematic antibiotics, were excluded; and (3) studies that did not report their results clearly, for example, reporting their results in figures only, were excluded.

### Study selection

Two reviewers (Chun-Ping Hao and Nan-Jue Cao) independently screened the titles and abstracts to identify potentially eligible studies. After screening the titles and abstracts, the full texts were reviewed for further screening (Chun-Ping Hao and Nan-Jue Cao). Any disagreements were discussed with the third author (Wei Wang). The agreement between the two investigators was assessed by the kappa test.

**Fig. 1:**
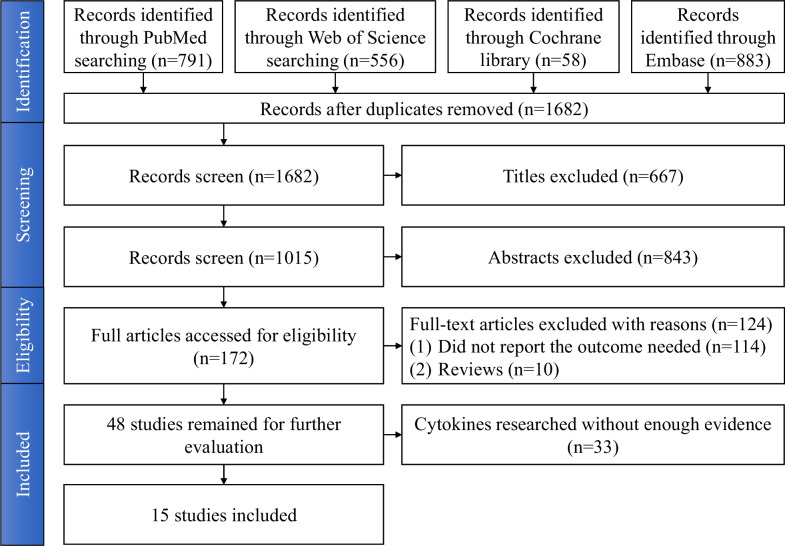
PRISMA flow diagram for studies selection process

Reference management software (Endnote X9) was used for the study screening process. The flow diagram according to the PRISMA guidelines is presented in Fig. 1.

### Data extraction

Two reviewers (Chun-Ping Hao and Nan-Jue Cao) independently extracted data from the eligible studies using a predesigned extraction form. Any disagreement was resolved by discussion with the third author (Wei Wang).

The extracted information included (1) first author’s name, country, publication year, study design; (2) gender of patients, mean age of patients; (3) definition of chronic periodontitis and smoking; (5) detection of cytokines from GCF and/or serum; (6) sample site for collecting the GCF, methods for measuring the cytokines; and (7) reported the cytokines by concentration and/or amount.

### Quality assessment

The risk of bias of the studies was assessed using different instruments according to the study design. Case–control studies and cohort studies were assessed by the Newcastle–Ottawa Scale (NOS) [33]. Studies were considered low quality with NOS scores of 0–4, moderate quality with scores of 5–7, and high quality with scores of 8–9. Cross-sectional studies were assessed by the quality form recommended by the Agency for Healthcare Research and Quality (AHRQ). Studies were defined as low quality (scored 0–4), moderate quality (scored 5–8), and high quality (scored 9–11) [34].

### Statistical analysis

Meta-analyses were performed with data extracted from at least three studies. Stata 14 software was used to perform the meta-analysis. The outcome was expressed as the standard mean difference (SMD) with 95% CI [35]. Heterogeneity was assessed by the Q-test and *I*^*2*^. Heterogeneity was assessed as low (*I*^2^ < 30%), moderate (*I*^2^ = 30–60%), and high (*I*^2^ > 60%) [35]. If *P* < 0.05 and *I*^2^ > 50%, the outcome was analysed by the random-effects model. Otherwise, a fixed-effects model was used [36–38]. Potential sources of heterogeneity may be the methods of cytokine measurement, publication year, and smoking status. Sensitivity analyses were carried out to assess the influence of single studies. Publication bias was assessed by Egger’s test [39]. A significant publication bias was considered if *P* < 0.05.

## Results

### Study selection

After searching four databases (Medline (PubMed), Cochrane Library, EMBASE, and Web of Science), we found 2288 potentially relevant publications. A total of 172 studies were selected for full-text review. A total of 124 studies were excluded during the full-text review stage. The remaining 48 studies were potentially eligible for the present meta-analysis. Then, we further conducted data extraction. Given the criteria of our meta-analysis, we only included cytokines reported by more than three studies. Therefore, after further assessment, 15 studies relating to 6 cytokines fulfilled the criteria of the current meta-analysis. A PRISMA flow diagram describing the process of the literature search and selection is presented in Fig. 1. The kappa value for the agreement between the two investigators was 0.80.

### Study characteristics

A total of fifteen studies were included in our manuscript, including eight cross-sectional studies and seven prospective cohort studies [20, 28, 40–52]. Their study characteristics are described in Additional file 2: Table S1. The criterion of the recruited chronic periodontitis patients of the included studies is various, however, according to the new classification system of periodontitis [9], the mainly recruited periodontitis patients are in stage III. Similarly, the definitions of smoking among the included studies varied. The most commonly considered aspects were the daily consumption of cigarettes and the duration of smoking. Many of the recruited smoking periodontitis patients have a history of consumption of ≥ 10 cigarettes/day. Therefore, many smoking periodontitis patients are in grade C. Most of the included studies employed enzyme-linked immunosorbent assay (ELISA) to detect cytokines; however, four recently published studies applied multiplex assay techniques to detect cytokines. Nine studies reported cytokines at the GCF level, while six studies reported cytokines at the serum level.

### Quality assessment

The quality assessments of the included studies are presented in Additional file 2: Table S1. Eight cross-sectional studies were assessed by AHRQ quality form and showed moderate to low quality. Seven prospective cohort studies were assessed by the NOS quality form, showing relatively high quality.

### Baseline GCF cytokine profile in chronic periodontitis patients with and without cigarette smoking

**Fig. 2:**
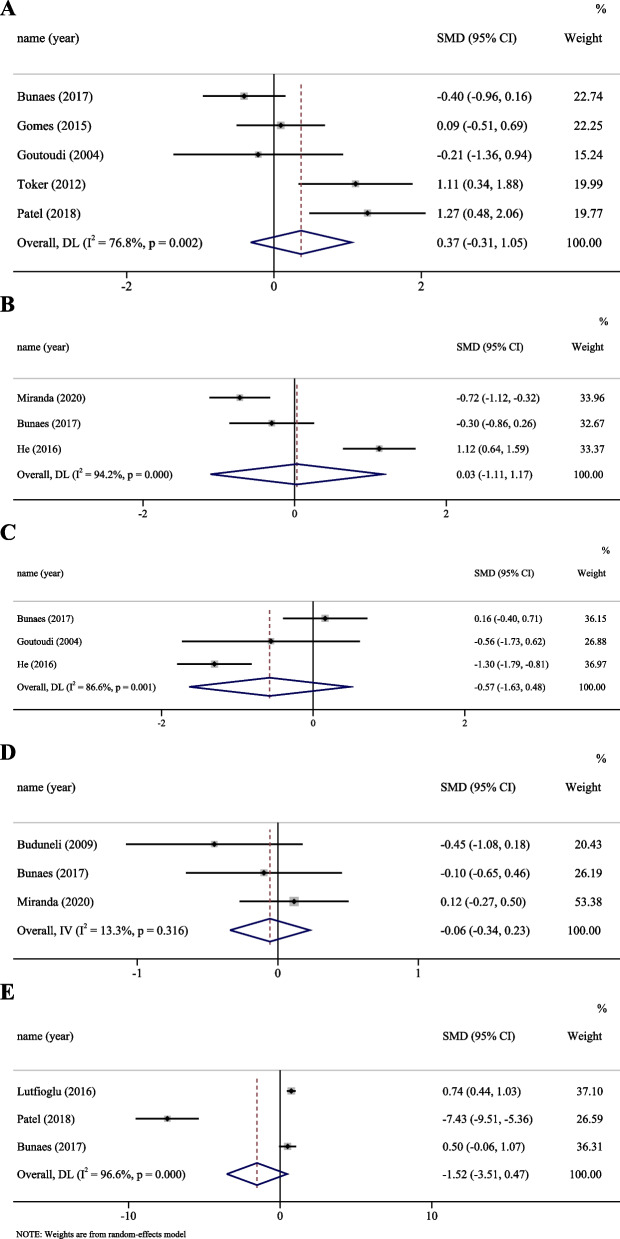
Meta-analysis for GCF cytokines in baseline: **A** IL-1β; **B** TNF-α; **C** IL-10; **D** IL-17; **E** IL-8

As shown in Fig. 2, nine studies [41–44, 47–51] compared the baseline cytokine profile in chronic periodontitis patients with and without smoking. Five cytokines were analysed at the GCF level in this meta-analysis, including IL-10, IL-1β, IL-8, IL-17, and TNF-α. The results showed that there were no significant differences in GCF cytokine levels between chronic periodontitis patients with and without smoking. Meta-analysis of four cytokines (IL-1β, TNF-α, IL-8, IL-10) showed high heterogeneity, while one cytokine (IL-17) showed low heterogeneity. Publication bias was assessed by Egger’s test (Additional file 2: Table S2). There was no significant publication bias.

### Baseline serum cytokine profile in chronic periodontitis patients with and without cigarette smoking

**Fig. 3:**
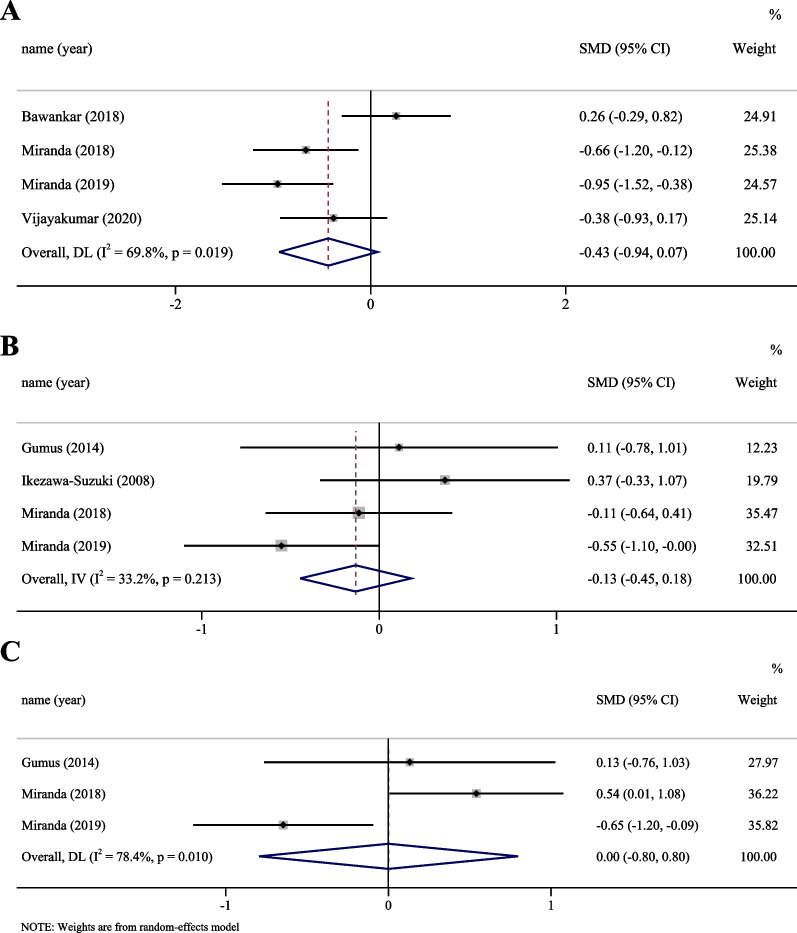
Meta-analysis for serum cytokines in baseline: **A** IL-1β; **B** TNF-α; **C** IL-6

Six studies reported the serum cytokine levels in patients with chronic periodontitis with and without smoking [20, 28, 40, 45, 46, 52]. At the serum level, three cytokines (IL-1β, IL-6, and TNF-α) were analysed. No significant difference was observed in the serum cytokine profile between the two groups of patients (Fig. 3). A high level of heterogeneity was observed in the meta-analysis of IL-1β and IL-6, while a low level of heterogeneity was observed for TNF-α. Publication bias assessed by Egger’s test showed no significant bias (Additional file 2: Table S2).

### GCF cytokine profile in chronic periodontitis patients with and without cigarette smoking following periodontal therapy

**Fig. 4:**
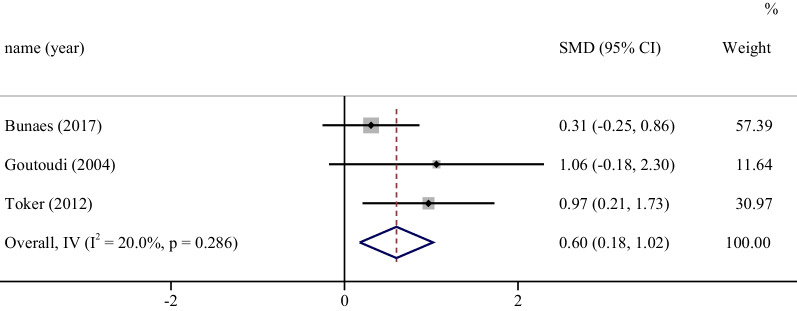
Meta-analysis for GCF cytokines after periodontal therapy: IL-1β

Three studies were included to compare the cytokine profile of the two groups of patients after periodontal therapy [41, 43, 47]. One cytokine (IL-1β) in the GCF reported by more than three studies was analysed by meta-analysis. The results showed that smoking patients with periodontitis had significantly higher IL-1β levels in the GCF after periodontal therapy than nonsmoking periodontitis patients (Fig. 4). This meta-analysis showed a low level of heterogeneity and no significant publication bias (Additional file 2: Table S2).

### Sensitivity analysis

The robustness of the meta-analysis for cytokines reported by more than three studies was tested by sensitivity analysis, which was conducted by systematically removing one study at a time and recalculating the pooled results. We did not observe a significant change after sensitivity analysis for IL-1β in GCF and TNF-α in serum. However, for IL-1β in serum, after omitting one study [40], the smoking patients with periodontitis showed significantly lower values than nonsmoking patients. The details are shown in Additional file 2: Table S3.

## Discussion

This meta-analysis explored the effect of smoking on the cytokine profile of patients with periodontitis. To comprehensively explore the influence of smoking on periodontitis, we synthesized the evidence to analyse the effect of smoking on the cytokine profile of periodontitis both in serum and GCF. The cytokine profile in GCF could reflect the local inflammatory state of periodontitis patients, while the cytokine profile in serum may indicate the systemic inflammatory situation. In addition, considering that smoking is reported to impair the effect of periodontal therapy [53], we also compared the cytokine profile of the two groups of patients after periodontal therapy to determine the effect of smoking on periodontal therapy.

We found that there was no significant difference both locally and systemically in the baseline cytokine profile between smoking and nonsmoking patients with periodontitis. Moreover, this meta-analysis found that periodontitis patients who smoked showed significantly higher IL-1β values in GCF than nonsmoking periodontitis patients after periodontal therapy. The periodontal therapy included in our meta-analysis is consisted of nonsurgical periodontal therapy and surgical periodontal treatment. This indicates that smoking may impair the response of periodontitis to periodontal therapy. This outcome is in accordance with other studies [54, 55]. A recently published meta-analysis demonstrated that smoking periodontitis patients showed less probing depth reduction and clinical attachment level gain than nonsmoking periodontitis patients after nonsurgical periodontal therapy [31]. Similar results were obtained by another meta-analysis reporting that smoking could compromise the clinical outcome of periodontal flap surgery [56].

IL-1β is a prominent proinflammatory cytokine in periodontitis produced by numerous cells, such as macrophages, neutrophils, lymphocytes, fibroblasts, and natural killer (NK) cells [57]. IL-1β plays a critical role in alveolar bone resorption. It can stimulate the production of prostaglandin E2 (PGE2) and matrix metalloproteinases (MMPs), degrading extracellular matrix proteins [58]. In addition, IL-1β regulates the balance between receptor activator of nuclear factor-kappa B ligand (RANKL) and Osteoprotegerin (OPG) to promote osteoclastogenesis, causing bone loss [59, 60]. Moreover, many clinical studies have found that IL-1β is closely associated with the progression of clinical attachment loss [61]. The level of IL-1β has great monitoring value in predicting the progression of periodontitis [62, 63].

In the present meta-analysis, we found that smoking periodontitis patients have significantly higher GCF IL-1β values than nonsmoking patients after periodontal therapy.

In the present meta-analysis, the included studies reported the level of cytokines in GCF by inconsistent methods, for example, concentration and/or total amounts. We included studies reporting cytokines by both methods, which is consistent with other meta-analyses [64, 65]. For studies reporting the quantity of cytokines by both the concentration and total amounts [41, 49–51], we adopted the data reported by concentration rather than the total amounts because the concentration is the reporting method adopted by most studies. Moreover, reporting results by concentration eliminated the influence of the GCF volume on the level of cytokines. It has been widely recognized that the GCF volume could be influenced by smoking habits and the periodontal pathogenesis state [66]. However, we still lack guidance for reporting GCF biomarkers. We are looking forward to a criterion to standardize reports about GCF cytokines. We conducted sensitivity analyses to eliminate the influence of these factors on the final outcome. We found no significant change in the conclusions after sensitivity analyses. In the future, a standard method for reporting GCF cytokines is required.

The strength of our meta-analysis is that although many studies have been devoted to determining the impact of smoking on periodontitis, the most common approach is to report the clinical outcome [67]. The underlying mechanism has been less demonstrated and remains unclear. Our meta-analysis synthesized evidence to research the influence of smoking on the cytokine profile of periodontitis, which fills the gap in the understanding of the mechanism by which smoking affects periodontitis. We found that smoking exerts a significant influence on the cytokine profile of periodontitis, providing evidence-based support to explain the mechanism by which smoking affects the immune response of periodontitis. Moreover, the outcome of the present meta-analysis indicates that we should pay more attention to smoking periodontitis patients after periodontal therapy. However, the outcome of our research also needs to be interpreted with caution due to high heterogeneity observed in some cytokines. Additional studies to more thoroughly address this issue are needed.

## Conclusion

There was no significant difference between periodontitis patients with and without smoking in the baseline cytokine levels. However, after periodontal therapy, smoking periodontitis patients showed significantly higher GCF IL-1β levels than nonsmoking periodontitis patients.

## Supplementary Information

**Additional file 1.** PRISMA Checklist.

**Additional file 2: Table S1.** Characteristics of included studies;** Table S2**. Publication bias of meta-analysis;** Table S3**. Sensitive analysis of meta-analysis with more than three included studies; Search strategy.

## Data Availability

All data generated or analysed during this study are included in this published article and its Additional file 1, Additional file 2.

## Abbreviations

GCF: Gingival cervical fluid
PROSPERO: International Prospective Register of Systematic Reviews
NFκB: Nuclear factor kappa B
MAPK: Mitogen activated protein kinase
PRISMA: Preferred Reporting Items for Systematic Review and Meta-Analysis
SD: Standard deviation
RCTs: Randomized controlled trials
SRP: Scaling and root planning
NOS: Newcastle–Ottawa Scale
AHRQ: Agency for Healthcare Research and Quality
SMD: Standard mean difference
ELISA: Enzyme-linked immunosorbent assay
NK: Natural killer
PGE2: Prostaglandin E2
MMPs: Matrix metalloproteinases
RANKL: Receptor activator of nuclear factor-kappa B ligand
OPG: Osteoprotegerin
