# Supplementary material for: The impact of smoking on periodontitis patients’ GCF/serum cytokine profile both before and after periodontal therapy: a meta-analysis
Source: BMC Oral Health. 2023 Feb 1;23:60. doi: 10.1186/s12903-023-02768-8 (PMC9893604; doi:10.1186/s12903-023-02768-8)
Supplement: Supplementary file 2 — Additional file 2: Table S1. Characteristics of included studies; Table S2. Publication bias of meta-analysis; Table S3. Sensitive analysis of meta-analysis with more than three included studies; Search strategy. [file 12903_2023_2768_MOESM2_ESM.docx]

The impact of smoking on periodontitis patients’ GCF/serum cytokine profile both before and after periodontal therapy: a meta-analysis

Chun-Ping Hao,^1,†^, Nan-Jue Cao,^1,†^, Yu-He Zhu,^1^, Wei Wang,^1,*^

^1^ School and Hospital of Stomatology, China Medical University, Liaoning Provincial Key Laboratory of Oral Diseases, Shenyang, Liaoning, Peoples R China

^†^ These authors contributed equally to this work.

* Correspondence should be addressed to Wei Wang; wwang75@cmu.edu.cn

**Catalogue**

**Supplementary Table 1:** Characteristics of included studies…………………………………………………………………………. Page 3

**Supplementary Table 2:** Publication bias of meta-analysis…………………………………………………………………………... Page 17

**Supplementary Table 3:** Sensitive analysis of meta-analysis with more than three included studies………………………………... Page 18

**Search Strategy:** ……………………………………………………………………………………………………………………… Page 19

Supplementary Table 1

| Author, year, country | Study design | Mean age of patients (mean±SD) | Gender of patients (M/F) | Definition of chronic periodontitis | Definition of smoking and nonsmoking | Sample site and collection method | Detection method | Quality assessment |
| --- | --- | --- | --- | --- | --- | --- | --- | --- |
| Bunaes, 2017, Norway | Prospective study | Smoker: 56.6±2.07 Nonsmoker: 57.9±1.86 | Smoker: 8/17  Nonsmoker: 13/12 | At least four non-adjacent teeth with interproximal probing depth ≥6 mm, clinical attachment loss ≥5 mm and bleeding on probing (BOP). | Smokers: reporting smoking >10 cigarettes/d for at least 5 years and with baseline pre-treatment (T0) serum cotinine level ≥300 ng/ml.  Non-smokers: reporting never or no smoking the last 5 years and with T0 serum cotinine level ˂15 ng/ml. | The deepest periodontal pocket in each participant was sampled.  The paper strips were placed 1–2 mm into the entrance of the pocket and left in place for 30 s.  Results reported by pg/ml and pg/30s. | Multiplex kit Bio-Plex Human Pro Assay for IL-1β, TNF-α, IL-10, IL-17, IL-8 in GCF. | Moderate |
| Gomes, 2015, Brazil | Prospective study | Smoker: 45.8±5.1  Nonsmoker: 47.3±6.7 | Smoker: 11/9  Nonsmoker: 10/13 | Presence of at least 12 teeth, excluding third molars; clinical diagnosis of biofilm-induced gingivitis and moderate-to-severe generalized chronic periodontitis; at least four teeth with PPD ≤ 5 mm and four teeth with PPD ≥6 mm, with supragingival biofilm, marginal bleeding, clinical attachment loss (CAL) and bleeding on probing (BOP). | Smokers: no description.  Non-smokers: never smoking. | GCF samples were harvested from four different sites drawn from 3–5 mm PPD sites and four from sites with 6–10 mm PPD per patient.  GCF samples were collected using Periopaper and assessed with a calibrated Periotron. Results reported by pg/µl. | ELISA for IL-1β in GCF | High |
| Goutoudi, 2004, Greece | Prospective study | 45.4 | 5/7 | More than 20 remaining teeth. Moderate to advanced periodontal disease as evidenced by multiple sites with a probing depth of 5 mm or more, extensive radiographic bone loss and bleeding on gentle probing. | NA | Probing depths ≥5 mm and a gingival index of 2 or 3 and were defined as diseased sites.  A sterile paper strip was inserted into the gingival crevice, until mild resistance was felt, and was kept there for 30 s.  Results reported by pg/µl. | ELISA for IL-1β, IL-10 in GCF | Moderate |
| Toker, 2012, Turkey | Prospective study | Smoker: 38.4±5.5  Nonsmoker: 38.7±5.9 | Smoker: 9/6  Nonsmoker: 7/8 | Periodontally diseased subjects were diagnosed with generalized chronic periodontitis (>30% of sites with a clinical attachment level and probing depth of ≥5 mm). | Smokers: regularly smoked ≥20 cigarettes/d.  Non-smokers: not having smoked cigarettes in their lifetime. | No description for sample site choosing.  A standard paper strip was placed in the pocket until mild resistance was felt and then left in place for 30 s.  Results reported by pg/site. | ELISA for IL-1β in GCF | Moderate |
| He, 2016, China | Cross-sectional study | Smoker: 46.5±8.3  Nonsmoker: 46.7±9.1 | Smoker: 26/12  Nonsmoker: 29/11 | The ChP was diagnosed by (1) presence of ≥2 interproximal sites with ≥3 mm clinical attachment loss, not on the same tooth, and (2) presence of ≥2 interproximal sites with ≥4 mm probing depth (PD) occurring at two or more different teeth, or presence of ≥5 mm PD of a site. | Smokers: patients smoked more than 10 cigarettes daily for longer than 2 years.  Nonsmokers: The patients who quit smoking for more than 10 years or had no smoking habits. | The GCF samples were taken from the deepest site of the PD.  Filter paper strips (2 mm × 10 mm) were placed at the bottom of the periodontal pocket and kept in place for 30 s to adsorb and collect the GCF. Four sites were selected, including the buccal side, far from the buccal side, the lingual side and far from the lingual side.  Results reported by ng/ml. | ELISA for TNF-α, IL-10 in GCF | Low |
| Gumus, 2014, Turkey | Cross-sectional study | 50.1±5.3 | 12/8 | Individuals had ≥4 teeth in each jaw with a probing depth (PD) of ≥5 mm, clinical attachment level (CAL) of ≥4 mm, and ≥50% alveolar bone loss at least in two quadrants. | Smokers: smoking ≥10 cigarettes/day for more than five years.  Nonsmokers: no history of tobacco smoking at all; former smokers had smoked <10 cigarettes/day for <5 years and had quit at least 6 months prior to inclusion in the study. | Five millilitres of venous blood were taken from the antecubital vein by a standard venipuncture method and serum was separated from blood by centrifugation at 1500 g for 10 min. The serum samples were then stored at -40℃ and thawed immediately before biochemical analyses. | ELISA for TNF-α, IL-6 in serum | Low |
| Ikezawa-Suzuki, 2008, Japan | Prospective study | Smoker: 51.8±2.2  Nonsmoker: 56.9±1.6 | Smoker: 8/4  Nonsmoker: 1/22 | NA | Smokers: daily smokers. All the smokers were cigarette smokers.  Nonsmokers: never smoked tobacco. | Serum was obtained from clotted venous blood samples, and centrifuged at 2000 g for 20 min and then stored at -80℃ until use. | ELISA for TNF-α in serum | Moderate |
| Miranda, 2018, Brazil | Cross-sectional study | Smoker: 51.4±7.9  Nonsmoker: 51.9±8.7 | Smoker: 9/17  Nonsmoker: 12/18 | Subjects were >30 years old, had generalized CP and at least 15 teeth, excluding third molars. Subjects were required to present >30% of sites with probing depth (PD) and clinical attachment level (CAL) ≥4 mm and bleeding on probing (BOP) and, a minimum of six teeth distributed in the four quadrants with at least one site with PD and CAL ≥5mm and BOP. The subjects were also required to have at least two teeth indicated to exodontia, due to advanced periodontitis (sites with PD and CAL ≥7mm with BOP, mobility and/or bone loss achieving more than half of the root). | Smokers: have smoked at least 10 cigarettes per day for at least the past 10 years.  Nonsmokers: never smoked. | Fasted peripheral blood samples were collected in the morning into an appropriate tube. Immediately after blood collection, the serum was separated from blood by centrifugation (10 min at 1,400 rpm, 4°C) and stored in aliquots at -80°C. | Multiplex immunoassay for TNF-α, IL-6, IL-1β in serum. | Moderate |
| Buduneli, 2009, Turkey | Prospective study | Smoker: 48±4.4  Nonsmoker: 44.54±5.53 | Smoker: 6/4  Nonsmoker: 4/6 | At least two interproximal sites with clinical attachment level (CAL) ≥4 mm and probing depth (PD) ≥5 mm not on the same tooth, two of which were in the anterior region. | Smokers: smoking ≥10 cigarettes/day for >5 years.  Nonsmokers: never smoked | GCF samples were obtained from the buccal aspects of four interproximal sites. Selection criteria for sampling sites were PD ≥5 mm, CAL ≥4 mm, and the presence of bleeding on probing in single-rooted teeth.  Filter paper strips were placed in the gingival sulcus/pocket for 30 seconds.  Results reported by pg/µl and pg/two samples. | ELISA for IL-17 in GCF. | Moderate |
| Miranda, 2019, Brazil | Cross-sectional study | Smoker: 51.1±7.7  Nonsmoker: 52.7±8.3 | Smoker: 10/17  Nonsmoker: 8/18 | Subjects with CP had generalized disease, defined as >30% of sites with concurrent probing depth (PD) and clinical attachment level (CAL) ≥4 mm and bleeding on probing (BOP), and a minimum of six teeth distributed in the four quadrants presenting at least one site with PD and CAL ≥5 mm and BOP. | Smokers: At least 10 cigarettes per day for at least the past 10 years.  Non-smokers: never smoked. | Fasted peripheral blood samples were collected in the morning, within 1 week after clinical examination, into an appropriate tube. Immediately after blood collection, the serum was separated by centrifugation (10 min at 1300 rpm, 4 °C) and stored in aliquots at −80 °C. | Multiplex fluorescent bead-based immunoassay for TNF-α, IL-6, IL-1β in serum. | Moderate |
| Lutfioglu, 2016, Turkey | Cross-sectional study | Smoker: 42.17±1.07  Nonsmoker: 45.13±1.85 | Smoker: 9/8  Nonsmoker: 7/8 | A diagnosis of generalized chronic periodontitis was based on radiographic evidence of alveolar bone and attachment loss, with probing pocket depths of ≥5 mm for at least eight teeth. | Smokers: smoked ≥15 cigarettes/d for ≥5 years  Nonsmokers: no previous history of smoking. | The deepest six sites of each subject were chosen for gingival crevicular fluid sampling.  Gingival crevicular fluid samples were collected using periopaper strips. The paper strip was inserted into the gingival crevice up to 1 mm, or until mild resistance was felt, and was left in place for 30 s.  Results reported by pg/ml and pg/30s. | ELISA for IL-8 in GCF | Low |
| Patel, 2018, India | Cross-sectional study | Smoker: 50.2±7.8  Nonsmoker: 60.2±8.7 | NA | 30% of sites with CAL and PD ≥5 mm. | Smokers: regularly smoked ≥20 cigarettes per day.  Nonsmokers: not having smoked one hundred or more cigarettes in their lifetime. | The test sites for GCF sample collection in the periodontitis patients comprised of five different nonadjacent sites that exhibited the deepest PD on clinical examination.  The GCF sample was collected by placing the 1–5μl calibrated volumetric microcapillary pipettes at the entrance of the gingival sulcus. The GCF sample was collected from all the five sites with a collection time of no more than 5 min from each site, and the collected sample was pooled to calculate the volume. Pooled GCF sample of 3μl was collected from all these sites.  Results reported by pg/µl. | ELISA for IL-1β, IL-8 in GCF. | Moderate |
| Miranda, 2020, USA | Cross-sectional study | Smoker: 51.7±8.5  Nonsmoker: 51.8±8.1 | Smoker: 10/16  Nonsmoker: 10/15 | >35 years old, ≥15 teeth (excluding third molars), >35% of sites with visible plaque and/or calculus, >30% of sites with probing depth (PD) and clinical attachment level (CAL) ≥4 mm and bleeding on probing (BOP), and ≥6 teeth with at least one site presenting CAL and PD ≥5 mm, distributed in the four quadrants. | Smokers: History of consumption of ≥10 cigarettes/day (moderate‐to‐high smokers) for at least the past 10 years (self‐reporting). Nonsmokers: No history of smoking. | Two non‐contiguous diseased sites (CAL and PD ≥5 mm with BOP) were randomly selected per patient for GCF sampling.  One standard paper strip was introduced ~2 mm into the sulcus/pocket for 30 s. Twenty seconds later, a second strip was inserted into the same sulcus/pocket for more 30 s.  Results reported by pg/site and pg/µl. | Multiplex fluorescent bead‐based immunoassay for IL-6, IL-17 in GCF. | Moderate |
| Bawankar, 2018, India | Cross-sectional study | NA | NA | Patients with untreated severe CP, as assessed by clinical finding of PPD ≥5 mm and CAL ≥5mm. (≥30% of teeth affected) and with radiographic evidence of bone loss. | Patients who were current smokers and with history of smoking at least 10 cigarettes per day for the last 3 years. | 5ml of venous blood sample was drawn in the morning between 9:00 and 11:00 am. Once collected, samples were allowed to clot at room temperature for 20 min. Then the clot was removed by centrifuging at 1500 g for 10 minutes. | ELISA for IL-1β in serum. | Low |
| Vijayakumar, 2020, India | Prospective study | NA | 52/0 | Attachment loss of ≥5 mm at more than 30% of the sites, bleeding on probing, and patients with ≥20 functional teeth. | Smokers are assessed based on smoking status by US Center for Disease Control and Prevention. | 5 mL venous blood sample was collected before SRP from the antecubital fossa venipuncture using the 23-gauge needle with 5-mL syringe. Then the blood was placed in the clot activator tube for 30 min and then centrifuged at 3000rpm for 10 min. Then the supernatant serum is separated from the clot activator tube. It was then transferred to a plastic vial and stored at –80ºC until the time of assay. | ELISA for IL-1β in serum. | Moderate |

Supplementary Table 2

| Biological fluid | | Cytokine | Number of studies | Sample Size | | Publication bias | | |
| --- | --- | --- | --- | --- | --- | --- | --- | --- |
|  |  |  |  | Smoke | Non-Smoke | Coefficient | SE | *P* |
| GCF | Baseline | IL-1β | 5 | 82 | 83 | 3.19 | 4.29 | 0.511 |
|  |  | TNF-α | 3 | 115 | 115 | 9.51 | 23.46 | 0.755 |
|  |  | IL-10 | 3 | 70 | 70 | 1.48 | 7.01 | 0.868 |
|  |  | IL-17 | 3 | 97 | 95 | -4.00 | 1.37 | 0.21 |
|  |  | IL-8 | 3 | 142 | 130 | -7.65 | 3.07 | 0.243 |
|  | After therapy | IL-1β | 3 | 47 | 45 | 2.52 | 1.81 | 0.397 |
| Serum | | TNF-α | 4 | 73 | 91 | 3.26 | 3.00 | 0.39 |
|  |  | IL-6 | 3 | 61 | 68 | 0.37 | 8.40 | 0.972 |
|  |  | IL-1β | 4 | 104 | 107 | -10.57 | 56.44 | 0.869 |

Supplementary Table 3

A: Sensitive analysis of IL-1β in GCF.

| Omitted study | Number of studies | Heterogeneity | | Standardized mean difference | | |
| --- | --- | --- | --- | --- | --- | --- |
|  |  | *I^2^* | *P* | SMD | 95% CI | *P* |
| Bunaes DF, 2017, Norway | 4 | 67% | 0.028 | 0.6 | (-0.09, 1.3) | 0.09 |
| Gomes SC 2015, Brazil | 4 | 82.2% | 0.001 | 0.45 | (-0.48, 1.38) | 0.343 |
| Goutoudi P 2004, Greece | 4 | 81.9% | 0.001 | 0.48 | (-0.31, 1.27) | 0.233 |
| Toker H, 2012, Turkey | 4 | 74.7% | 0.008 | 0.18 | (-0.54, 0.91) | 0.621 |
| Patel RP, 2018, India | 4 | 69.8% | 0.019 | 0.14 | (-0.52, 0.81) | 0.669 |

B: Sensitive analysis of TNF-α in serum.

| Omitted study | Number of studies | Heterogeneity | | Standardized mean difference | | |
| --- | --- | --- | --- | --- | --- | --- |
|  |  | *I^2^* | *P* | SMD | 95% CI | *P* |
| Gumus P, 2013, Turkey | 3 | 52% | 0.124 | -0.17 | (-0.5, 0.17) | 0.328 |
| Ikezawa-Suzuki I, 2008, Japan | 3 | 1.9% | 0.361 | -0.26 | (-0.61, 0.09) | 0.15 |
| Miranda TS, 2018, Brazil | 3 | 55.4% | 0.106 | -0.14 | (-0.53, 0.25) | 0.473 |
| Miranda TS, 2019, Brazil | 3 | 0 | 0.553 | 0.07 | (-0.31, 0.45) | 0.722 |

C: Sensitive analysis of IL-1β in serum.

| Omitted study | Number of studies | Heterogeneity | | Standardized mean difference | | |
| --- | --- | --- | --- | --- | --- | --- |
|  |  | *I^2^* | *P* | SMD | 95% CI | *P* |
| Bawankar PV, 2018, India | 3 | 1.1% | 0.364 | -0.66 | (-0.98, -0.34) | 0 |
| Miranda TS, 2018, Brazil | 3 | 77.7% | 0.011 | -0.36 | (-1.04, 0.33) | 0.307 |
| Miranda TS, 2019, Brazil | 3 | 65% | 0.057 | -0.26 | (-0.8, 0.27) | 0.333 |
| Vijayakumar S, 2020, India | 3 | 79.8% | 0.007 | -0.45 | (-1.16, 0.26) | 0.215 |

**Search Strategy**

**Embase**

#9 #7 OR#8

#8 Cytokine OR (chemotactic AND cytokine) OR chemokine OR (marker, AND biological) OR (biological AND marker) OR (biologic AND marker) OR (biological AND markers) OR biomarker OR (immune AND markers) OR (marker, AND clinical)

#7 ‘cytokine’/exp OR ‘chemokine’/exp OR ‘biological marker’/exp

#6 #4 OR #5

#5 Periodontitides OR (periodontitis, AND adult) OR (chronic AND periodontitides) OR (diseases, AND periodontal) OR (periodontal AND disease)

#4 ‘periodontitis’/exp OR ‘chronic periodontitis’/exp OR ‘aggressive periodontitis’/exp OR ‘periodontics’/exp OR ‘periodontal disease’/exp

#3 #1OR #2

#2 Smoking AND behaviors OR (behavior, AND smoking) OR (smoking AND habit) OR (habit, AND smoking) OR (smoking AND habits) OR (smoking, AND tobacco) OR (smoking, AND cigarette) OR smoke

#1 ‘smoke’/exp OR ‘smoking’/exp OR ‘hookah’/exp OR ‘cigarette smoking’/exp OR ‘cigar smoking’/exp OR ‘pipe smoking’/exp

**Web of Science**

**#1 cytokines** (Topic) or **chemokines** (Topic) or **biomarkers** (Topic) or **cytokine** (Topic) or **chemotactic cytokine** (Topic) or **chemokine** (Topic) or **marker, biological** (Topic) or **biological marker** (Topic) or **biologic marker** (Topic) or **biological markers** (Topic) or **biomarker** (Topic) or **immune markers** (Topic)

#2 **periodontitis** (Topic) or **chronic periodontitis** (Topic) or **aggressive periodontitis** (Topic) or **periodontics** (Topic) or **periodontal diseases** (Topic) or **periodontal attachment loss** (Topic) or **chronic periodontitides** (Topic) or **periodontoses** (Topic) or **diseases, periodontal** (Topic) or **periodontal disease** (Topic)

#1 **smoking behaviors** (Topic) or **smoking** (Topic) or **tobacco smoking** (Topic) or **cigarette smoking** (Topic) or **cigar smoking** (Topic) or **smoking habit** (Topic) or **habit, smoking** (Topic) or **smoking, tobacco** (Topic) or **smoking, cigar** (Topic) or **smoking, cigarette** (Topic) or **smoke** (Topic)

#3 and #2 and #1

**Cochrane Library**

#1 MeSH descriptor: [chronic periodontitis] explode all trees

#2 chronic periodontitis

#3 (periodontitis):ti,ab,kw OR(periodontities):tiab,kw OR (chronic periodontitis):ti,ab,kw OR(adult periodontitis):ti,ab,kw OR (periodontitis):ti,ab,kw

#4 MeSH descriptor: [periodontitis] explode all trees

#5 #1OR #2 OR #3OR #4

#6 MeSH descriptor: [Tobacco Smoking] in all MeSH products

#7 MeSH descriptor: [Smoke] in all MeSH products

#8 (smoking):ti,ab,kw OR (smoking behaviors):ti,ab,kw OR(tobacco smoking):ti,ab,kw OR (behavior,smoking):ti,ab,kw OR(smoking habit):ti,ab,kw

#9 #6 OR #7 OR #8

#10 MeSH descriptor: [Cytokines] explode all trees

#11 MeSH descriptor: [chemokines] explode all trees

#12 (cytokine):ti,ab,kw OR (cytokine, chemotactic):ti,ab,kw OR (biologic marker):ti,ab,kw OR (biomarker):ti,ab,kw OR (cytokines, chemotactic):ti,ab,kw

#13 #10 OR #11 OR #12

#5 AND #9 AND #13

**Medline (PubMed)**

(((((((((((((((((((Smoke[MeSH Terms]) OR (Smoking[MeSH Terms])) OR (Smoking Water Pipes[MeSH Terms])) OR (Tobacco Smoking[MeSH Terms])) OR (Cigarette Smoking[MeSH Terms])) OR (Cigar Smoking[MeSH Terms])) OR (Pipe Smoking[MeSH Terms])) OR (Smoking Behaviors)) OR (Behavior, Smoking)) OR (Smoking Habit)) OR (Habit, Smoking)) OR (Smoking Habits)) OR (Smoking, Tobacco)) OR (Smoking Pipe)) OR (Waterpipes, Smoking)) OR (Smoking, Cigar)) OR (Smoking, Cigarette)) OR (Smoking, Pipe)) AND (((((((((((((Periodontitis[MeSH Terms]) OR (Chronic Periodontitis[MeSH Terms])) OR (Aggressive Periodontitis[MeSH Terms])) OR (Periodontics[MeSH Terms])) OR (Periodontal Diseases[MeSH Terms])) OR (Periodontal Attachment Loss[MeSH Terms])) OR (Periodontitides)) OR (Periodontitis, Adult)) OR (Chronic Periodontitides)) OR (Periodontitis, Prepubertal)) OR (Periodontoses)) OR (Diseases, Periodontal)) OR (Periodontal Disease))) AND (((((((((((((cytokines[MeSH Terms]) OR (Chemokines[MeSH Terms])) OR (biomarkers[MeSH Terms])) OR (Cytokine)) OR (Chemotactic Cytokine)) OR (Chemokine)) OR (Marker, Biological)) OR (Biological Marker)) OR (Biologic Marker)) OR (Biological Markers)) OR (Biomarker)) OR (Immune Markers)) OR (Marker, Clinical))
